# Insights into Hemodynamic Features of Survivors and the Deceased with Acute Brain Injury: A Step Forward Tailored Treatment

**DOI:** 10.3390/jcm12124021

**Published:** 2023-06-13

**Authors:** Hanna Miszczenkow, Łukasz Krzych

**Affiliations:** 1 Department of Anesthesiology and Intensive Care, School of Medicine in Katowice, Medical University of Silesia, Medyków 14, 40-752 Katowice, Poland; 2 Department of Cardiac Anesthesia and Intensive Care, Silesian Centre for Heart Diseases, Marii Skłodowskiej-Curie 9, 41-800 Zabrze, Poland

**Keywords:** subarachnoid hemorrhage, hemodynamical monitoring, pulmonary artery catheter

## Abstract

Background: Pulmonary artery catheters are widely used for hemodynamical monitoring in critically ill patients. Acute brain injury is among the severe conditions treated in an intensive care unit. The advanced monitoring of hemodynamical parameters, fluid balance and adequate administered treatment based on those values are components of goal-directed therapy. Methods: A prospective observational study included adult patients who were hospitalized in the ICU due to acute bran injury, excluding brain oedema after cardiac arrest. Each patient had PAC inserted and hemodynamic data were collected during the first 3 days of the ICU stay every 6 h. Patients were divided into two groups based on the endpoint: the survivors and the deceased. Results: Length of stay in hospital differed between patiens. All patients, regardless of their outcome, had noradrenaline administered. The initial values of PAP differed between the groups (*p* = 0.05). There were positive correlations noticed between noradrenaline dose, CVP and fluid balance when compared to PCWP in a group of survivors and a positive correlation in the fluid balance when compared to PAP and PVRI. Lactate serum concentrations presented a correlation with the dose of noradrenaline in both groups. Conclusions: Upon acute brain injury, values of PVRI and PAP increase. This is corelated with fluid load and worsened by an excessive fluid treatment in the case of an inconsiderate approach for stabilizing the patient hemodynamically. PAC may present limited advantages in terms of PAP and PVRI control during the treatment.

## 1. Introduction

Any acute brain injury, either traumatic or non-traumatic, may lead to serious clinical consequences, including brain oedema, blood–brain barrier disorder or brain tissue ischemia. Brain tissue destruction, not only in its anatomical but also harmful and functional sense, plays a significant role in disturbing homeostasis in the face of decreased oxygen delivery [1]. In that aspect, it is highly essential to provide adequate cerebral blood flow, given what an oxygen-dependent organ the brain is. Hemodynamic stabilization in acute illness of the brain plays an important role due to the association of mean arterial pressure, stroke volume effects, and cerebral blood flow [2]. The optimization of hemodynamic features of the circulatory system is also one of the major preventive measures against delayed cerebral ischemia. In order to avoid such complications, cerebral blood flow must be increased so the “hyperdynamic” strategy to increase CI (cardiac index) or the “hypertension” approach to affect MAP are useful [3].

Over the years, especially due to findings in critically ill patients suffering from septic shock, new possibilities were established to obtain effective clinical practice equipment. A more practical and less invasive approach to hemodynamic monitoring, but still an accurate estimation of patient’s condition, was the priority. The starting point to all those considerations was the arterial blood pressure waveform and Poiseuille’s law. Thank to the analysis of left ventricular stroke volume, numerous vascular resistance and vascular compliance monitoring systems based on various algorithms were introduced to clinical use. Saugel et al. [4] comprehensively discusses that issue by presenting models such as the two-/three-/four-element Windekessel models or the Modelflow technique, as well as the Hemac method along with calibration and thermodilution that became the base for invasive and minimally invasive hemodynamic monitoring methods.

Such monitoring has been successful and proved to be useful in a group of fragile patients suffering from severe heart disease. While PAP is known as an independent risk factor for one-month mortality among these patients, it has been shown that monitoring as in PICCO, where ELWI is estimated, may enable an accurate decision in regard to fluid therapy as well as antidiuretic drugs [5].

Finally, the wide use of ultrasound technology is also helpful and promising for fluid therapy and fluid responsiveness when the inferior vena cava (IVC) is measured. That also puts the emphasis on the goal-tailored therapy of the critically ill. One must remember that the situation differs if the patient is mechanically ventilated or not; it is called IVC distensibility or collapsibility in each situation, respectively [6,7].

Providing an advanced hemodynamical monitoring system using a pulmonary artery catheter and introducing a tailored treatment with fluids, vasoconstrictors and inotropes should increase the chances of patients surviving with acute brain injury by maintaining an optimal perfusion.

The aim of this study is to assess the hemodynamic profile of patients with acute brain injury and to make a comparison between the survivors and the deceased. This profound insight may serve as the first step in applying a tailored hemodynamic treatment among this unique group of critically ill patients.

## 2. Materials and Methods

**Scheme 1:**
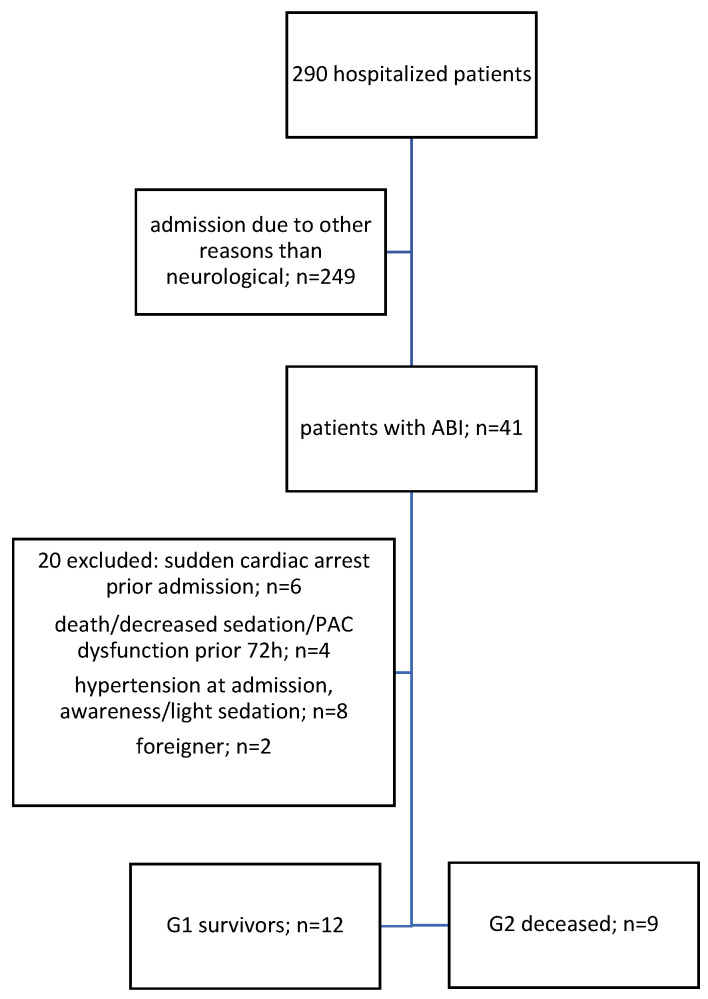
Flowchart of the study population.

This prospective observational study included adult patients admitted to the intensive care unit (ICU) from February 2021 to February 2022 due to an acute brain injury (ABI). The study received formal approval of the Ethics Committee of the Medical University of Silesia in Katowice, Poland (KNW/0022/KB/203/19). The inclusion criteria were: age >18 years, acquired ABI of any origin (traumatic or non-traumatic), neurological status requiring deep sedation assessed as −5 with the RASS (Richmond Agitation Sedation Scale), and acute respiratory failure requiring mechanical ventilation. The exclusion criteria were: BMI < 18.5 kg/m^2^ or BMI > 35 kg/m^2^, history of chronic obstructive pulmonary disease, severe heart failure (NYHAIII/NYHA IV), chronic kidney disease, hypertension requiring the constant infusion of hypotensive agent on admission, sudden cardiac arrest prior to admission, previous surgery (other than neurosurgical) in the immediate period before ICU admission, death in the first three days upon ICU admission, and unknown legal status of the patient. The patients’ flowchart is depicted on Scheme 1.

All patients required advanced hemodynamic monitoring due to signs of acute hemodynamic instability. The hemodynamic monitoring was performed using a pulmonary artery catheter (PAC). All PACs (MeritMedical Criticath) were inserted through a standardized procedure performed by an experienced intensivist via the right internal jugular vein using the MeritMedical Percutaneous Sheath Introducer 8.5F ExactaTM under real-time ultrasonography guidance after the sterile preparation of the site of catheterization. Once the catheter was inserted, measurements of all available hemodynamic data were performed. The software Patient Monitor Mindray BeneView T8 automatically calculated the values of hemodynamic data. Three samples of blood were retrieved (i.e., arterial, venous, mixed) and analyzed using SIEMENS Rapidpoint 500 (Erlangen, Germany). The PAC measurements were repeated 4 times a day (i.e., every 6 h). The observation time was 72 h; so, overall 12 time points were defined for every patient. Additionally, doses of any catecholamine use were noted and fluid balance was counted at each of the twelve time points.

Statistical analysis was performed using MedCalc Statistical Software version 18.2.1 (MedCalc Software, Ostend, Belgium; 2018). Quantitative variables were expressed using medians with their interquartile ranges (IQR). The type of distribution of the variables was verified with the Shapiro–Wilk test. Qualitive variables were presented using absolute values and percentages. The differences between groups were calculated with Student’s *t*-test or the Mann–Whitney *U* Test. The Fisher exact test was applied for categorical data. Correlation was assessed using Spearman’s rank correlation test. A *p*-value < 0.05 was considered significant.

## 3. Results

The study group comprised 21 subjects, including 14 men (67%). There were 12 survivors (G1 group) and nine patients died (G2 group). Patients suffered from SAH (subarachnoid hemorrhage), and postoperative and postinjury intracranial hematoma. There were statistically significant differences between survivors and the deceased in terms of age, BMI, APACHE II score and total length of stay in hospital. To the contrary, there was no difference in length of stay in ICU (*p* = 0.29). No difference between groups was observed in SOFA scores and the duration of mechanical ventilation. Other compared variables did not present any statistical differences. No difference was found between groups in regard to initial noradrenaline dosage. Adrenaline was administered depending on the hemodynamic profile (CI), according to the judgment of the intensivist. However, no statistical differences were recorded, but in the group of survivors the administration of adrenaline was quicker (42% vs. 11%) and the initial dose was higher than in the group of deceased. The lack of statistical significance may result from the fact that those two groups were not numerous enough and that cases would need further investigation. Basic demographic and clinical data are depicted in Table 1.

**Figure 1:**
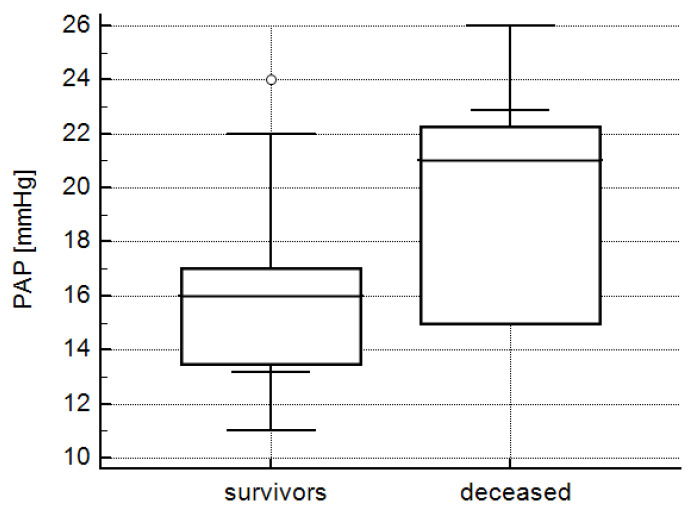
Initial values of PAP in G1 and G2 in G1.

The starting point was to measure the initial values of the three-day hemodynamic parameters monitored regularly. The first outline is presented in Table 2. Patients who died initially were characterized by higher values of PAP: 16 vs. 21 mmHg; *p* = 0.05 (Figure 1).

Moreover, moving forward to three-day-long observation, the groups were comparable in terms of the applied hemodynamic support to restore and maintain hemodynamic equilibrium (Table 3). No differences in neither catecholamine dose nor fluid load were found.

Table 4 presents correlations between the hemodynamic profile and the applied treatment in survivors during a three-day observation period. There was a statistically significant positive correlation between fluid load and PCWP (R = 0.64, *p* = 0.03). Moreover, there was a positive correlation between three-day noradrenaline dose and CVP (R = 0.73, *p* = 0.007).

**Figure 2:**
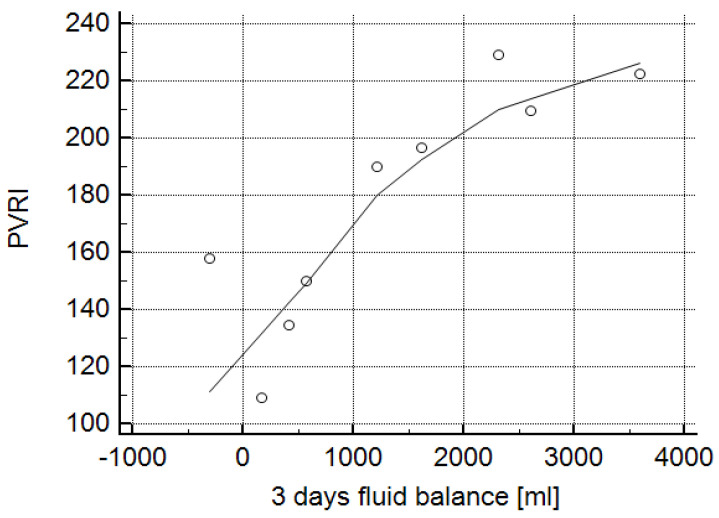
Three-day fluid balance—PVRI correlation in G2.

**Figure 3:**
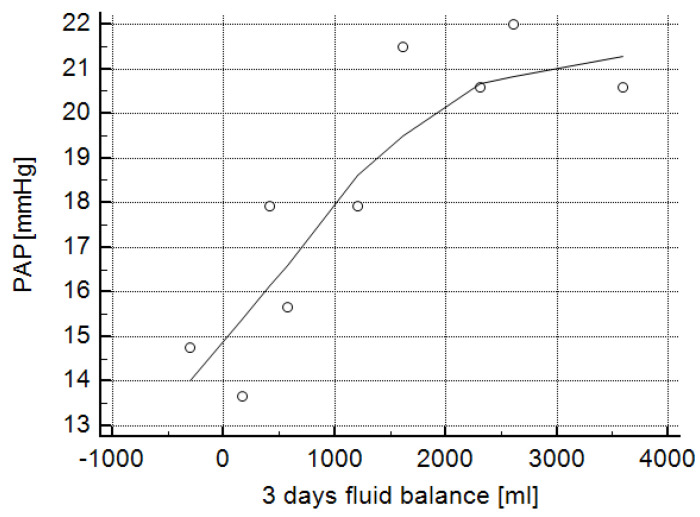
Three-day fluid balance—PAP correlation in G2.

Although in G1 there was a correlation between noradrenaline and CVP, in G2, such a correlation was not observed. Despite that, we found correlations between fluid load, and PAP and PVRI. In the deceased, PVRI and PAP positively correlated with a cumulative three-day fluid balance, as presented in Table 5 and Figure 2 and Figure 3.

By analyzing microcirculation indicators, positive statistically significant correlations were found in terms of noradrenaline dose and lactate concentration in the group of survivors as well as in the group of deceased. Lactate concentration increased when the dosage of noradrenaline rose. That regularity was observed in G1 (R = 0.81, *p* = 0.001) as well as in G2 (R = 0.73, *p* = 0.02) (Table 6 and Table 7). Microcirculatory differences were noticed when each day of observation was calculated separately. There was a correlation between lactate concentration and noradrenaline dose on day 1 (R = 0.79, *p* = 0.002) and day 2 (R = 0.62, *p* = 0.03). In the G2 group, this result is only noticeable only on day 3 (R = 0.81, *p* = 0.008).

None of the variables, neither the hemodynamic nor microcirculation variables, were helpful for the prediction of death because all AUC oscillated circa 0.5–0.6 with *p* > 0.05.

## 4. Discussion

The aim of the study was to assess the hemodynamic profile of patients hospitalized in our ICU due to acute brain injury and investigate whether PAC is an optimal tool to conduct hemodynamical monitoring in that group of patients.

Both groups, survivors and deceased, have had catecholamines administered: either noradrenaline or/and adrenaline.

The worldwide accepted clinical standard is based on maintaining an accurate CPP. This is achieved not only by drugs preventing vasospasm such as nimodipine, or intravascular procedures, but also by keeping an appropriate MAP. Both groups had catecholamines administered. In our study, there were no statistically significant differences in cumulative three-day noradrenaline dose with the initial dose on the first day. Similar results achieved in a pilot trial by Gathier et al. [8] show that there is no reason in increasing the noradrenaline dose with the goal of inducing hypertension because it is not associated with any benefits; rather, it increased serious adverse effects two-fold. Monitoring ought to be focused on either the invasive or noninvasive monitoring of potential vasospasm. Another study looking at the effects of excessive blood pressure conducted by Derkwah Oppong et al. [9] revealed that tailored treatment with appropriate ICP was a “game changer” and reduced the incidence of vasospasm by a factor of two. Mean systolic blood pressure was higher in the group with vasospasm and arbitrary MAP ≥ 95 mmHg was found to be an independent factor of generally poor outcomes or DCI (delayed cerebral ischemia). Our survey proved that uncritically increased doses of noradrenaline are burdened with an unfavorable endpoint. MAP must not be increased at all costs.

It was noticeable in our trial that the group of survivors (G1) received adrenaline faster than the deceased (G2), although no statistically significant difference was found. Due to the limitations of the study, it is impossible to draw conclusions. Nevertheless, this aspect remains an interesting topic for future research. Currently, there is not enough evidence in the medical literature to draw any conclusions.

Moreover, in our group, the monitoring of vasospasm was not equal for all patients. TCCD or CT procedures were performed selectively and based on clinical indicators.

The deceased group was characterized by higher values of PAP at the initial point of the trial. Moreover, three-day cumulative data presented a positive correlation between fluid balance, and PVRI and PAP (R = 0.85, *p* = 0.004 and R = 0.86, *p* = 0.003, respectively).

These findings confirm the effects of the pathophysiology of neurogenic pulmonary edema (NPE), which is found in approximately 50% of cases of SAH [10]. Three main systematic changes occurred, such as vasoconstriction, hypertension and increased preload. Additionally, sympathetic agitation induced increased blood flow from systemic high resistance vessels to low resistance pulmonary vessels. Therefore PCWP, PAP and PVRI increased. Moreover, according to the humoral theory, the brain injury initiates bursts of endogenic catecholamines that harm the basement of the alveolar epithelium and cause the leakage of proteins and blood cells into the alveolar space [11]. Numerous surveys confirm that liberal fluid therapy is associated with vasopressors followed by worse clinical outcomes, especially neurological outcomes, as well as increased threats of cardiovascular and pulmonary complications [12,13,14].

Excessive fluid load along with sympathetic system activation worsens the primary condition of the overload of pulmonary capillaries [15,16]. That association was confirmed in our survey.

Among other findings, a positive correlation between serum lactate concentration and noradrenaline in both groups in a three-day observation was found. Lozano et al. also found evidence that high serum lactate concentrations are associated with poor outcomes in critically ill patients as a reflection of impaired tissue metabolism, perfusion and adrenergic stimulation [17,18] Changes in metabolic and inflammatory status have been reported after TBI and appear to be linked, not only just after the incident, but also spread over time, even months after the incident [19]. The very first catecholamines that are involved in the brain–heart axis are noradrenaline and adrenaline [20]. It must be reiterated that the treatment must be adjusted precisely to the patient. In a survey by Son et al. [21], researchers proved that both primary increased cerebrospinal fluid lactate levels were significantly higher and serum lactate levels were relatively high in the poor neurologic outcome group at each time point after cardiac arrest. In another trial, Annan et al. [22], exploring the aspect of lactate and lactate dehydrogenase in carotid cistern, concluded that increased levels of these two factors may be early biomarkers of brain injury after SAH and may be useful for predicting delayed cerebral ischemia. Changes in lactate level do not only result due to the usage of catecholamines, but also due to an injured brain itself. Especially important are glial cell astrocytes, which are susceptible to noradrenaline and produce lactate in brain tissue [23]. Serum lactate levels also release lactate from the injured brain into the blood, and that release is dominant over lactate uptake after TBI by the brain [24].

Speaking of the usefulness of lactate, it is widely recognized as an indicator of metabolic changes due to decreased oxygen delivery. Although it has a stable position in clinical practice, in the literature, the role of another clinical indicator is emphasized.

Capillary refill time (CRT) may seem to be an old-fashioned physical examination. One must remember, however, that it might be helpful and should not be omitted due to its susceptibility to microcirculatory alterations which are so frequent among the critically ill, when even a single-spot prolonged CRT might be independently associated with abnormal microcirculation and increased mortality [25].

A systematic review and meta-analysis by Putowski et al. presented a summary of 10 studies and 917 patients where CRT was evaluated in comparison with the MAP in septic patients. The review’s conclusion was that achieving an MAP > 65 mmHg does not guarantee the normalization of CRT in that group of patients, which indicates a poor correlation between MAP and CRT. Nevertheless, researchers still point out that this assessment ought to be proceeded with fluid therapy [26].

In a big set of data collected from five countries and 28 care intensive care units, the ANDROMEDA-SHOCK trial showed that among patients with septic shock, a resuscitation strategy targeting the normalization of capillary refill time, compared with a strategy targeting serum lactate levels, did not reduce all-cause 28-day mortality. However, only peripheral perfusion-targeted resuscitation was associated with less organ dysfunction at 72 h when patients received less resuscitation fluids within the first 8 h [27]. This opens up possibilities of an easy, bed-side option of care and quick assessment. Other researchers have also taken that issue under consideration. In an observational study about the significance of the CRT examination site, despite healthy volunteers being enrolled in the trial, CRF was found to be the optimal fast method in deciding whether to administer fluids as it reacts rapidly to peripheral perfusion changes. It seems to be a valuable indicator with or without the company of others, i.e., lactate concentration measurements [28].

The further finding was a positive correlation between PCWP and fluid balance in G1 (R = 0.63, *p* = 0.03). Mutoh et al. [29] proposed a strategy to achieve goal-directed fluid therapy in SAH patients based on transpulmonary hemodynamic monitoring. Their results revealed a much more extensive fluid load than in our survey’s three-day observation of 248.3 (129.4–348.2) mL/d. In Mutoh’s trial, the daily fluid intake gradually increased uniformly to 2661 ± 547 mL/d, with a range from 1516 to 3807 mL/d until day 3. Although differences in fluid treatment approaches are present in both studies, both surveys did not find statistically significant differences between the patient groups—in our trial, between the survivors and the deceased, and in Mutoh’s trial, among patients with different clinical grades according to the severity of their clinical condition. Upon researching available databases, no clear reason for this result can be found.

Nevertheless, fluid load may increase CI, which was shown by Kurtz et al. [30], but still, one has to bear in mind that avoiding pulmonary complications is one of the goals of the treatment.

The length of stay of our patients was also statistically different in the case of the group of survivors, though LOS in the ICU did not present any differences in the between-group comparison.

This result is in agreement with the outcome of work by Martini et al. [31] where investigators explored the association between fluid balance and outcomes after SAH. The same conclusion was also presented by Fu et al. [32]. Here, on the other hand, the impact of lactate serum was examined and it has been shown that patients with lower lactate concentrations stayed longer in the hospital. Moreover, other researchers only proved their correlation based on the total length of stay, and not the length of stay in the ICU.

Currently, there are not many studies that have investigated the usefulness of pulmonary artery catheters in patients suffering from acute brain injury. PAC had its time, but was systematically replaced by other methods. Still, it is the only method of estimating PCWP and PAP; however, for many years, the theory has suggested the administration of fluid therapy, which has been questioned according to its indicators [33]. American data show the faltering interest in PAC for many researchers and clinicians in many patients’ groups, such as SAH patients, since the 2000s [34,35]. Other, less invasive methods play a greater role and transpulmonary thermodilution is currently one of the more popular methods [3,36]. In that perspective, PAC is useful only in the sublime population of critically ill. Yet, as Messina’s et al. research shows, the approach of treatment varies across the globe.

## 5. Limitations

There are a few possible limitations for our study. First, the size of the observed groups and relatively short duration that lasted on average 5 years may cause limitations in the calculations. This is an exploratory study. We prospectively included all research in a definite time period with a prespecified and limited budget. No a priori sample size calculation was possible due to the novelty of our study. However, a posteriori estimations, based on Spearman’s rank correlation coefficients, with an alpha of 0.01, let us assume that our study group was sufficiently large to draw reliable conclusions. Calculations suggested to enroll between 10 and 16 patients, depending on which correlation result was taken under consideration. Second, the etiopathogenesis of patients’ conditions differed. That made the group heterogenic in regard to the neurologic reason of admission to the ICU. Third, due to medical center limitations, patients did not have ICP measured by intracranial/intraventricular catheters. Fourth, the device for hemodynamic monitoring was not provided for continuous measurement. Another, fifth limitation or potential source of bias was that any intervention based on hemodynamic data was left to the judgment of the intensivist who took care of a patient. Moreover, not all patients had TCCDs, head CTs with contrast or echocardiograms performed. Due to these numerous limitations, further surveys are indispensable.

## 6. Conclusions

Patients with acute brain injury present numerous changes in their hemodynamical status. Any brain injury is a condition that requires widened monitoring of a patient because of its life-threatening capability. A pulmonary artery catheter is useful for showing the changes in pulmonary vessel resistance and pressure that occurs due to acute brain injury. Upon acute brain injury, values of PVRI and PAP increase. This is corelated with fluid load and is worsened by an excessive fluid treatment in the case of an inconsiderate approach to stabilize the patient hemodynamically. Patients enrolled to the survey had an adequate treatment provided according to hemodynamic monitoring; however, the hemodynamic profile cannot be the only useful indicator for possible outcomes. There are other factors that determine survival among patients with acute brain injury such as the vasoconstriction of brain arteries or cardiopulmonary complications that were not regularly monitored with TCCD or echocardiography, respectively, unless it was clinically necessary. PAC may present limited advantages regarding PAP and PVRI control during the treatment.

## Figures and Tables

**Table 1:** Basic characteristics of the study group with between-group comparisons.

Variable	Total	Survivors	Deceased	*p*
Male, *n* (%)	14 (66.67%)	8 (66.57%)	6 (66.67%)	1
Age (year), IQR	48 (44–59)	45 (38–52)	63 (48–65)	0.02
BMI (kg/m^−2^), IQR	25.71 (22.9–27.6)	26.21 (23.5–27.6)	24.4 (21.2–26.3)	0.04
BSA (m^2^), IQR	1.90 (1.8–2.1)	1.9 (1.8–2.1)	1.9 (1.6–2.06)	0.96
Length of stay in total, days, IQR	26 (18–29)	32 (19–58)	18 (14–26)	0.03
Length of stay in ICU, days, IQR	18 (10–22)	19 (16–24)	15 (9–29)	0.29
Diuretics *n* (%)	9; 42.8%	6; 50%	3; 33.33%	0.66
Sequential Organ Failure Assessment (SOFA), points, IQR	10 (8–11)	9.5 (8–11)	10 (9–11)	0.62
Acute Physiology and Chronic Health Evaluation II (APACHE II) points, IQR	15 (12–20.25)	13 (12–17.5)	20 (14.5–25.25)	0.04
Mechanical ventilation, days, IQR	13 (10.75–18.25)	12.5 (11.5–18)	15 (9–18.5)	0.94
Number of patients with norepinephrine on 1st day *n* (%)	20; 95%	11; 92%	9; 100%	1
Norepinephrine on the 1st day mean dose (ug/kg/min), IQR	0.2 (0.07–0.3)	0.17 (0.06–0.3)	0.21 (0.08–0.3)	0.8
Number of patients with adrenaline on the 1st day *n* (%)	6; 29%	5; 42%	1; 11%	0.18
Adrenaline on the 1st day mean dose, IQR	0 (0–0.1)	0 (0–0.023)	0 (0–0)	0.23
Hg 1st day (g/dL), IQR	11.45 (10.9–12.7)	11.53 (11.1–12.5)	11.38 (10.8–12.7)	0.73
Hematocrit % 1st day, IQR	34 (32.8–37)	34.01 (32.9–36.6)	33.9 (31.7–37)	0.76
Fluid balance (mL) after 1st day, mean per day, IQR	146.25 (276.23–412.5)	149.063 (273.12–415.9)	72.5 (276.3–341.3)	0.83
IVC distensibility, %	22.76 (16.49–28.67)	21.71 (15.29–26.82)	24.24 (20.67–31.66)	0.23

**Table 2:** Baseline hemodynamic profile (first assessment).

Variable	Total	Survivors	Deceased	*p*
PAP	17(15–21)	16 (13.5–17)	21 (15–22)	0.05
MAP	87 (80–99)	86.5 (81.5–99)	89 (79–104)	0.4
PCWP	10 (7–13)	12 (7.5–14.5)	8 (7–12)	0.4
SVRI	1790 (1504–2255)	1870 (1551–2490)	1774 (1504–2121)	0.7
PVRI	208 (45–331)	53 (30–295)	229 (198–221)	0.1
CI	3.6 (2.8–4.1)	3.3 (2.5–3.8)	3.9 (3.2–4.4)	0.2
CVP	7.12 (6.33–7.92)	7.13 (6.33–7.75)	7.35 (5.56–9.42)	0.72

PAP: pulmonary artery pressure, MAP: mean arterial pressure, PCWP: pulmonary capillary wedge pressure, SVRI: systemic vascular resistance index, PVRI: pulmonary vascular resistance index, CI: cardiac index, CVP: central venous pressure.

**Table 3:** Applied hemodynamic support.

Variable	Total	Survivors	Deceased	*p*
Three-day norepinephrine dose (mcg/kg/min), IQR	0.15 (0.05–0.3)	0.13 (0.04–0.3)	0.21 (0.09–0.3)	0.52
Three-day epinephrine dose (mcg/kg/min), IQR	0 (0–0.005)	0 (0.0–0.009)	0 (0–0)	0.24
Three-day fluid balance (mL), IQR	248.3 (129.4–348.2)	233.1 (77.9–347.9)	250.7 (179.5–348.8)	0.47

**Table 4:** Correlations between hemodynamic data, and catecholamines and fluid balance in the G1 survivors group (three-day cumulative data).

Variable	MAP	CVP	PCWP	CI	SVRI	PVRI	PAP
Fluid balance	rho = −0.18 *p* = 0.59	rho = 0.24 *p* = 0.445	rho = 0.64 *p* = 0.03	rho = 0.38 *p* = 0.23	rho = −0.34 *p* = 0.29	rho = −0.04 *p* = 0.91	rho = 0.41 *p* = 0.18
NorA	rho = −0.13 *p* = 0.68	rho = 0.73 *p* = 0.007	rho = 0.537 *p* = 0.07	rho = 0.46 *p* = 0.14	rho = −0.203 *p* = 0.527	rho = −0.21 *p* = 0.51	rho = 0.06 *p* = 0.86
A	rho = 0 *p* = 1	rho = 0.15 *p* = 0.64	rho = −0.05 *p* = 0.89	rho = −0.34 *p* = 0.28	rho = 0.281 *p* = 0.377	rho = −0.20 *p* = 0.53	rho = −0.05 *p* = 0.13

MAP: mean arterial pressure, CVP: central venous pressure, PCWP: pulmonary capillary wedge pressure, CI: cardiac index, SVRI: systemic vascular resistance index, PVRI: pulmonary vascular resistance index, PAP: pulmonary artery pressure, NorA: noradrenaline, A: adrenaline.

**Table 5:** Correlations between hemodynamic data, and catecholamines and fluid balance in the deceased G2 group (three-day cumulative data).

Variable	MAP	CVP	PCWP	CI	SVRI	PVRI	PAP
Fluid balance	rho = 0.27 *p* = 0.67	rho = 0.07 *p* = 0.86	rho = 0.52 *p* = 0.15	rho = 0.167 *p* = 0.67	rho = −0.25 *p* = 0.52	rho = 0.85 *p* = 0.004	rho = 0.86 *p* = 0.003
NorA	rho = 0.07 *p* = 0.87	rho = −0.35 *p* = 0.36	rho = −0.55 *p* = 0.13	rho = 0.4 *p* = 0.29	rho = −0.22 *p* = 0.58	rho = −0.517 *p* = 0.154	rho = −0.29 *p* = 0.46
A	rho = 0.27 *p* = 0.48	rho = 0.07 *p* = 0.86	rho = 0.14 *p* = 0.73	rho = 0.27 *p* = 0.48	rho = −0.27 *p* = 0.48	rho = 0.27 *p* = 0.48	rho = 0.55 *p* = 0.12

MAP: mean arterial pressure, CVP: central venous pressure, PCWP: pulmonary capillary wedge pressure, CI: cardiac index, SVRI: systemic vascular resistance index, PVRI: pulmonary vascular resistance index, PAP: pulmonary artery pressure, NorA: noradrenaline, A: adrenaline.

**Table 6:** Mean three-day comparison of microcirculatory variables in correlation with catecholamines and fluid balance for group G1 (survivors).

Variable	Lactate	pCO2 gap	SvO2
Fluid balance	rho = 0.53 *p* = 0.08	rho = 0.09 *p* = 0.76	rho = −0.35 *p* = 0.27
NorA	rho = 0.81 *p* = 0.001	rho = −0.04 *p* = 0.91	rho = 0.13 *p* = 0.68
A	rho = 0.24 *p* = 0.45	rho = 0 *p* = 1	rho = 0.41 *p* = 0.18

NorA: noradrenaline, A: adrenaline, SvO2: mixed blood saturation.

**Table 7:** Mean three-day comparison of microcirculatory variables in correlation with catecholamines and fluid balance for group G2 (deceased).

Variable	Lactate	pCO2 gap	SvO2
Fluid balance	rho = −0.3 *p* = 0.43	rho = −0.38 *p* = 0.31	rho = −0.57 *p* = 0.11
NorA	rho = 0.73 *p* = 0.02	rho = −0.02 *p* = 0.97	rho = 0.42 *p* = 0.27
A	rho = 0.41 *p* = 0.27	rho = −0.55 *p* = 0.13	rho = 0.14 *p* = 0.73

NorA: noradrenaline, A: adrenaline, SvO2: mixed blood saturation.

## Data Availability

Data are available from the authors of the study.

## Abbreviations

A—adrenaline	ICU—intensive care unit
ABI—acute brain injury	IQR—interquartile range
AUC—area under curve	LoS—length of stay
BMI—body mass index	MAP—mean arterial pressure
BSA—body surface area	NorA—noradrenaline
CBF—cerebral blood flow	PAC—pulmonary artery catheter
CI—cardiac index	PAP—pulmonary artery pressure
CPP—cerebral perfusion pressure	PCWP—pulmonary capillary wedge pressure
CT—computed tomography	PVRI—pulmonary vascular resistance index
CVP—central venous pressure	SvO2—mixed blood saturation
G1—survivors group	SAH—subarachnoid hemorrhage
G2—deceased group	SVRI—systemic vascular resistance index
Hg—hemoglobin	TCCD—transcranial color doppler.
ICP—intracranial pressure	
